# Large saccular aneurysms of the vertebrobasilar arteries with segmental chronic mural ectasia: A case report and review of management strategies

**DOI:** 10.5339/qmj.2026.46

**Published:** 2026-06-22

**Authors:** Khaled Zammar, Omar Mohammed Alhatou, Abeer Safan, Majd Aboualrob, Mohammad Alhatou, Yahia Imam

**Affiliations:** 1 Neurology Department, Hamad General Hospital, Hamad Medical Corporation, Doha, Qatar; 2 Royal College of Surgeons in Ireland, Busaiteen, Bahrain; 3 Weill Cornell Medicine, Doha, Qatar; 4 Faculty of Medicine, Qatar University, Doha, Qatar

**Keywords:** Aneurysm, saccular, basilar, artery, ectasia, Qatar

## Abstract

**Background:**

Large saccular aneurysms of the basilar artery associated with chronic mural ectasia are rare and difficult to manage because of their high risk of thrombus formation and rupture. We present a case that highlights the complexities involved in the diagnosis and treatment decision-making of this condition.

**Case Presentation:**

A 54-year-old male with hypertension and ischemic cardiomyopathy presented to the Emergency Department via emergency medical services with confusion and unresponsiveness. Neuroimaging revealed a large basilar artery aneurysm with a mural thrombus and posterior circulation infarcts. Despite conservative management and planned angiography, the patient’s condition rapidly deteriorated, and he was declared brain dead within 36 h.

**Discussion:**

This case underscores the difficulties associated with managing basilar artery aneurysms, particularly in patients with mural ectasia. It examines the rationale for the initial conservative treatment and evaluates the potential benefits of early endovascular intervention. Literature insights are integrated to explore the implications for vessel integrity, thromboembolic risk, and intervention timing.

**Conclusion:**

This case highlights the urgent need for standardized guidelines for the management of basilar artery aneurysms. Future research should focus on comparing surgical and endovascular approaches, assessing long-term aneurysm progression, and developing risk-based treatment protocols.

## 1. INTRODUCTION

A saccular aneurysm, also known as a “berry aneurysm,” is characterized by the focal dilatation of a blood vessel with a rounded sac-like shape, usually arising at sites of arterial bifurcation due to weakened walls of the vessels.^1^ Large saccular aneurysms, which are more than 10 mm in diameter, are relatively less common in the basilar artery and often pose significant diagnostic and management challenges.^2^ Chronic mural ectasia involves diffuse dilatation and thinning of the vessel wall, which can be dangerous in many instances and carries a high risk of thrombus formation and rupture.^3^

Stroke and cerebrovascular diseases remain the leading causes of disability and mortality worldwide. Stroke is broadly classified into ischemic stroke, caused by vascular occlusion or thromboembolism, and hemorrhagic stroke, resulting from vessel rupture. Clinical practice varies depending on healthcare infrastructure and resource availability.^4,5^

This case was managed at Hamad Medical Corporation (HMC), the main public tertiary care provider in Doha, Qatar, which serves as the national referral center for acute stroke care. Qatar has undergone rapid healthcare development, and its public health system is consistently ranked among the most advanced in the Middle East due to sustained investment and alignment with international standards.^4,5^ In particular, HMC has implemented a comprehensive national stroke program that adheres to American Heart Association and European Stroke Organization recommendations, supported by 24/7 neuroimaging and endovascular services.^6^

The coexistence of saccular aneurysms with segmental chronic mural ectasia in the posterior circulation presents unique diagnostic and therapeutic challenges. This report describes a case encountered at HMC and reviews the available management strategies. It is prepared in accordance with the CARE (CAse REport) guidelines. Written informed consent for publication was obtained from the patient’s next of kin. Institutional approval was also secured from the Medical Research Center at HMC (MRC approval reference number: MRC-04-24-183).

## 2. CASE PRESENTATION

A 54-year-old Bangladeshi right-handed male presented to the Emergency Department via emergency ambulance services after being found by his roommates confused and lying on the floor at home approximately 5 h prior to arrival. His medical history, obtained from electronic medical records of previous encounters at HMC, included hypertension, ischemic cardiomyopathy with a low ejection fraction of 23%, and dyslipidemia. These are major cardiovascular risk factors that predispose to both atherosclerotic disease and thromboembolic complications, potentially contributing to aneurysm formation and progression. On examination, his vital signs were temperature 36.8°C (axillary), heart rate 72 beats per minute (bpm), respiratory rate 16 breaths per minute, blood pressure 187/126 mmHg, and oxygen saturation (SpO2) 99%. His National Institutes of Health Stroke Scale (NIHSS) was 5, indicating moderate stroke severity (the NIHSS ranges from 0 to 42, with scores of 1–4 indicating minor stroke, 5–15 moderate stroke, and >15 severe stroke). His deficits included impaired orientation (incorrect responses to month and age questions), partial gaze palsy, partial hemianopia, minor facial paralysis, and inattention. He was awake and alert to name, but inattentive and not following commands. Language assessment showed he could say a few sentences but required repeated stimulation to follow simple commands. Cranial nerve examination showed equal and reactive pupils (3 mm), intact extraocular movements, symmetric facial features, and a midline tongue. Motor assessment was limited due to patient non-compliance, but he moved all extremities spontaneously and against gravity. Sensory response was intact to pain, and no nystagmus or appendicular ataxia was noted. Trauma findings included a left frontal head laceration, anterior tongue bite, and urinary incontinence.

**Figure 1.**
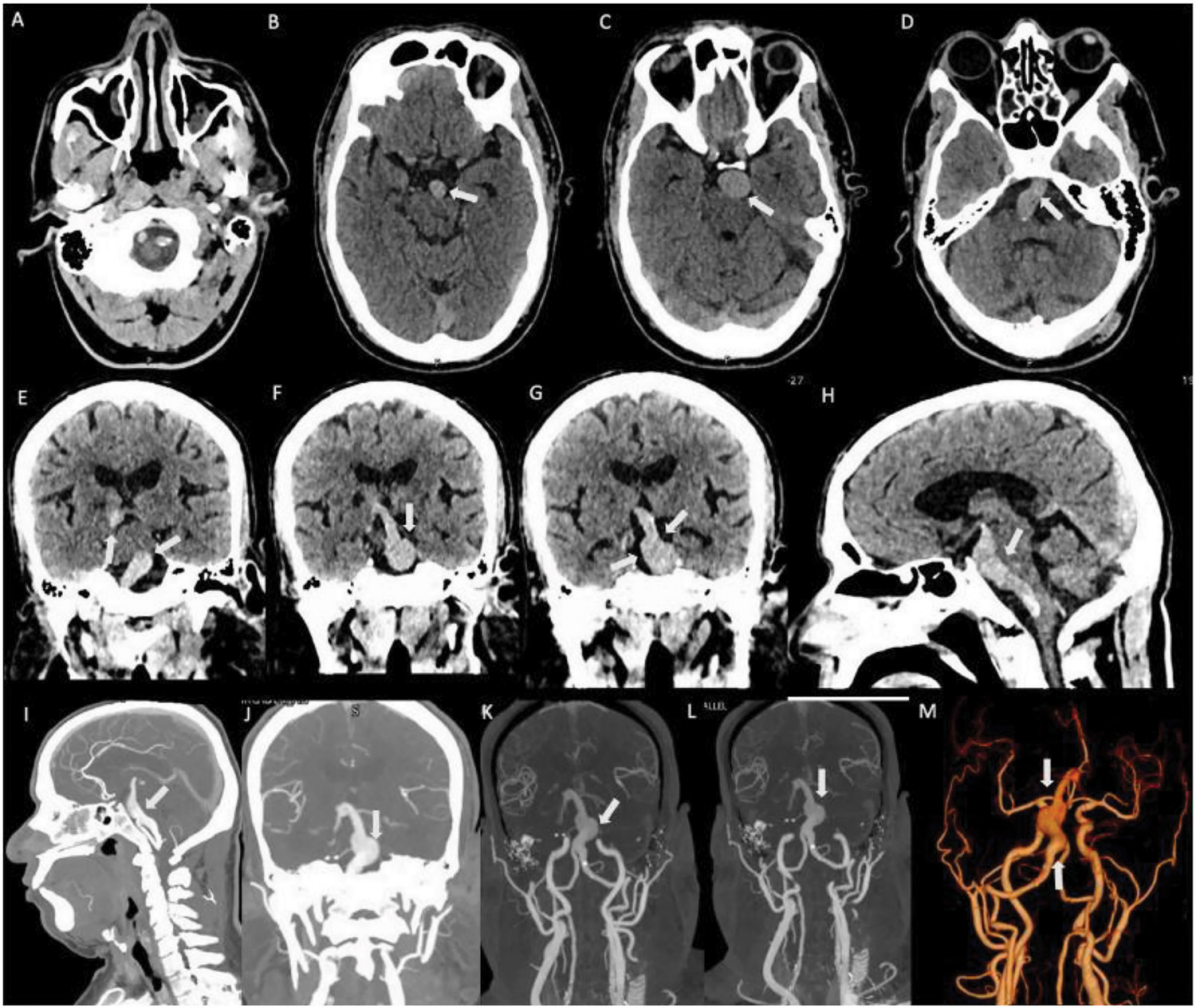
Computed tomography (CT) scan of the head and CT angiogram of the head and neck. (A–D) Non-contrast head CT scan showing the saccular dilatation of the basilar artery trunk. (E–M) CT angiogram of the head with intravenous (IV) contrast showing the aneurysmal dilatation of the basilar artery trunk.

Electrocardiogram (ECG) showed sinus rhythm with multiple premature ventricular contractions (PVCs), and a repeat ECG revealed bigeminy of PVCs. Stroke protocol was activated. Computed tomography (CT) of the head showed no acute intra- or extra-axial hemorrhage (Figure 1). There were bilateral periventricular hypodensities suggestive of chronic white matter microangiopathy, along with an old lacunar infarct in the right basal ganglia. The scan also demonstrated age-related involutional changes and encephalomalacia in the left occipital region. No midline shift, mass effect, or hydrocephalus was noted, and the basal cisterns were preserved. No craniofacial fractures were identified. A dilated left vertebral artery and basilar artery were also observed. CT perfusion revealed a perfusion defect in the bilateral parieto-occipital lobes, indicating an ischemic infarct without penumbra. CT cerebral angiogram showed a dilated, tortuous left vertebral artery (8.8 mm) and basilar artery (10.1 mm), suggestive of aneurysmal dilatation with partial thrombus in the distal basilar artery aneurysm, small-caliber bilateral posterior cerebral arteries (PCAs), and atherosclerotic calcifications in the vertebrobasilar circulation without significant stenosis or occlusion in the intracranial carotid arteries.

Laboratory results showed a white blood cell count of 8.6x109/L (reference range: 4.0–11.0x109/L), red blood cell count of 6.3x1012/L (reference range: 4.7–6.1x10¹²/L), hemoglobin of 16.2 g/dL (reference range: 13.8–17.2 g/dL), international normalized ratio of 1.1 (reference range: 0.8–1.2), urea of 6.7 mmol/L (reference range: 2.5–7.8 mmol/L), creatinine of 135 µmol/L (reference range: 62–115 µmol/L), sodium of 142 mmol/L (reference range: 135–145 mmol/L), potassium of 3.6 mmol/L (reference range: 3.5–5.1 mmol/L), troponin T of 27 ng/L (reference range: <14 ng/L), total cholesterol of 3.5 mmol/L (reference range: <5.2 mmol/L), triglycerides of 1.1 mmol/L (reference range: <1.7 mmol/L), high-density lipoprotein (HDL) cholesterol of 1.0 mmol/L (reference range: >1.0 mmol/L), low-density lipoprotein (LDL) cholesterol of 1.9 mmol/L (reference range: <3.0 mmol/L), and beta-hydroxybutyrate of 0.62 mmol/L (reference range: 0.0–0.6 mmol/L). Venous blood gas (VBG) analysis showed a pH of 7.34 (reference range: 7.32–7.42), PCO2 of 51 mmHg (reference range: 40–50 mmHg), HCO3- of 27 mmol/L (reference range: 22–28 mmol/L), and lactate of 2.7 mmol/L (reference range: 0.5–2.2 mmol/L).

Standard door-to-needle and door-to-reperfusion times were not applicable in this case, as the patient presented approximately 5 h after symptom onset (outside the thrombolysis window), and CT perfusion demonstrated a completed infarct with no salvageable penumbra. Furthermore, the underlying pathology was aneurysm-related thromboembolism rather than a primary occlusive stroke amenable to standard reperfusion therapy. The patient was initially hemodynamically unstable, with labile blood pressure, and was admitted to the medical intensive care unit 1 h after presentation, with plans for cerebral angiography using digital subtraction angiography once stabilized. The delay in performing angiography was necessitated by the patient’s hemodynamic instability and significant cardiac comorbidity (ejection fraction 23%), which posed substantial procedural risks. However, within 12 h of presentation, he deteriorated clinically, with a significant drop in Glasgow Coma Scale (GCS) score from 15 to 11, prompting an urgent diffusion-weighted magnetic resonance imaging (MRI) of the brain.

**Figure 2.**
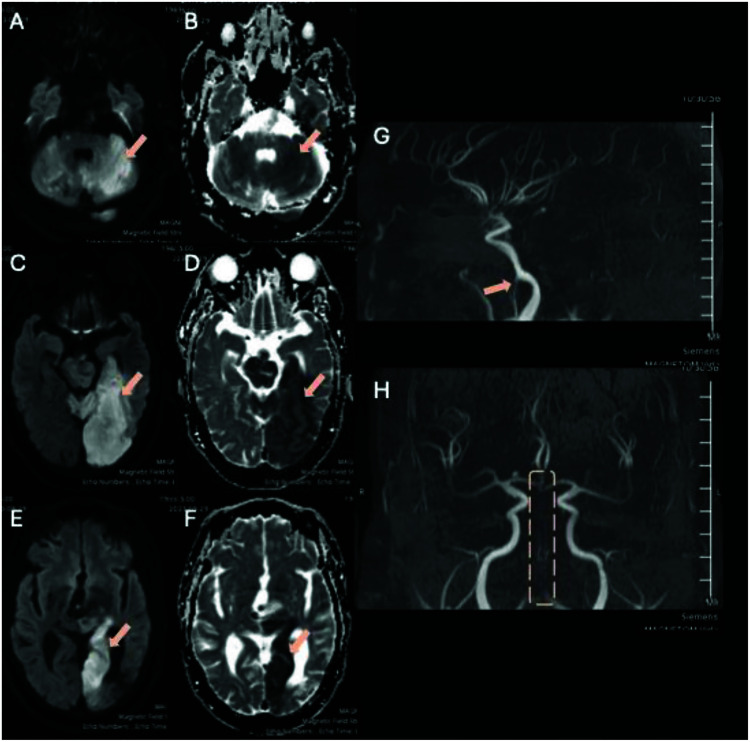
Magnetic resonance imaging (MRI) of the brain with magnetic resonance angiography (MRA). (A, C, E) Brain MRI showing a large area of diffusion restriction in the left occipital lobe extending into the temporal lobe and left thalamus, along with bilateral cerebellar infarcts, more prominent on the left, with involvement of the vermis. (B, D, F) Corresponding findings are seen on the apparent diffusion coefficient (ADC) maps. (G, H) Cranial MRA showing non-visualization of the distal vertebral arteries, basilar artery, and posterior cerebral arteries (PCAs), with preserved visualization of the distal internal carotid arteries (ICAs), anterior cerebral arteries (ACAs), and middle cerebral arteries (MCAs).

MRI revealed a large area of diffusion restriction in the left occipital lobe extending into the temporal lobe and left thalamus, along with bilateral cerebellar infarcts, more prominent on the left, with involvement of the vermis and corresponding changes on apparent diffusion coefficient (ADC) mapping (Figure 2). Cranial magnetic resonance angiography (MRA) showed non-visualization of the distal vertebral arteries, basilar artery, and PCAs. In contrast, the distal internal carotid arteries (ICAs), anterior cerebral arteries (ACAs), and middle cerebral arteries (MCAs) were adequately visualized.

Despite aggressive medical management, the patient’s condition continued to deteriorate. His blood pressure remained uncontrolled despite intravenous (IV) labetalol and antihypertensive medications administered via a nasogastric tube. Approximately 36 h after the initial presentation, he was declared brain dead.

## 3. DISCUSSION

This case is unique because of the uncommon combination of a large saccular aneurysm of the basilar artery and segmental chronic mural ectasia, which significantly complicated both diagnosis and treatment decisions. The management challenges were further heightened by the patient’s rapid neurological deterioration despite initial conservative treatment, emphasizing the urgent need for standardized guidelines for managing such complex cerebrovascular pathologies.

Moreover, the management of such cases presents significant challenges and requires a multifaceted approach. The basilar artery, being a critical vessel that supplies blood to the brain trunk and cerebellum, necessitates a comprehensive understanding and strategic planning for effective management.^3,7^ Several management strategies are available, including surgical intervention, endovascular treatment, and observation, each with its own set of indications and risks.

Surgical clipping remains a conventional strategy for the treatment of basilar artery aneurysms; however, its use in the presence of mural ectasia is limited due to the complexity of aneurysm access and the risk of postoperative complications.^7^ The involvement of cranial nerves and the delicate nature of brain trunk structures pose additional operational risks. Surgical treatment may be considered when the aneurysm displays signs of rupture or an imminent risk of rupture, and when the anatomical configuration allows safe access.^8^

On the other hand, endovascular management has gained substantial traction in the treatment of basilar artery aneurysms, especially because of advances in technology and technique. Embolization techniques have evolved, enabling the treatment of complex aneurysms through both direct and indirect approaches.^9^ The endovascular approach is particularly beneficial in cases where surgical clipping poses a significant risk because of the anatomical challenges associated with chronic mural ectasia.

Studies have shown that endovascular treatment can achieve favorable outcomes with lower morbidity rates compared to traditional surgery.^8,9^

Endovascular treatment, such as flow-diverting stents or coiling, should be considered and is generally preferred as it has lower complication rates and is more suitable for challenging locations such as the basilar artery.^10,11^

Another remarkable aspect of management involves observational strategies, especially for asymptomatic patients or those with non-complex aneurysms associated with mural ectasia, in whom the natural history of the disease may not justify aggressive intervention. The decision to adopt an attentive waiting approach is often guided by the patient’s risk factors, as well as the size and morphology of the aneurysm and its potential for natural regression.^12^ Longitudinal studies suggest that certain basilar trunk aneurysms may remain stable over time, supporting the observation strategy in selected cases.^13^

In addition, the presence of segmental chronic mural ectasia raises concerns about the potential for progressive disease, complicating the management decision-making process. Mural ectasia may indicate a broader underlying vascular pathology, requiring careful monitoring with regular imaging studies to evaluate changes in aneurysm size or configuration.^8^ The integration of multidisciplinary approaches, including neurology, vascular surgery, and interventional radiology, is essential for adapting specific management strategies to individual patient profiles and aneurysm characteristics.

In our patient, the additional complication of segmental chronic mural ectasia compromised vessel wall integrity and could necessitate surgical treatment. Cerebral angiography ensures a correct diagnosis of the aneurysm’s anatomy and helps in the selection of the appropriate treatment modality to achieve the optimal outcomes in such complicated cases.^10,11^ Initial conservative management consisted of intensive care unit admission for close neurological monitoring, aggressive blood pressure control with IV labetalol and oral antihypertensives, and hemodynamic optimization given his underlying cardiomyopathy. Digital subtraction angiography was planned once adequate hemodynamic stability was achieved. However, despite these measures and the plan for further evaluation, the patient rapidly deteriorated within two days. This tragic outcome underlines the unpredictable clinical course of large basilar artery aneurysms with mural ectasia, in which even short delays in intervention may result in disastrous complications.

Finally, the treatment of large saccular basilar artery aneurysms complicated by segmental chronic mural ectasia requires careful assessment of surgical, endovascular, and observational strategies. Although surgical clipping may be indicated in certain high-risk scenarios, endovascular techniques offer innovative solutions with promising outcomes. Additionally, the observation strategy is a valid approach for specific patient subsets. Ultimately, the complexity of basilar artery anatomy and the nuances of mural ectasia require an individualized treatment plan informed by ongoing research and clinical experience.^9^

## 4. CONCLUSION

This case underlines a gap in the literature and the need for future research on aneurysms with chronic mural ectasia and their management. Despite activation of the stroke protocol, conservative stabilization, and planned angiography, the patient’s condition rapidly deteriorated, and he was declared brain dead within 36 h of admission. The unfavorable outcome illustrates the difficulty of balancing conservative and early interventional approaches in such complex cases. Future studies on this topic should focus on establishing evidence-based guidelines for the optimal management of large saccular basilar artery aneurysms with chronic mural ectasia. Ideally, future studies should be randomized trials assessing the efficacy and safety of the various types of endovascular and surgical interventions, primarily focusing on long-term outcomes of aneurysm stability, thromboembolic events, rupture rates, and patient survival.

## DATA AVAILABILITY

All data generated or analyzed during this study are included in this published article. Further details are available from the corresponding author upon reasonable request.

## ETHICAL CONSIDERATIONS

Written informed consent was obtained from the patient for the publication of this case report and any accompanying images. Institutional ethical approval was not required as per local guidelines for case reports.

## AUTHOR CONTRIBUTION

KZ: Writing the manuscript. OMA: Literature review and writing the manuscript. AS: Review and editing. MA: Review and editing and image acquisition. MA: Supervision. YI: Supervision and review.

## FUNDING

The author(s) received no financial support for this article.

## ACKNOWLEDGMENT

The authors would like to thank the patient and his family for consenting to share the details of this case for academic and scientific purposes.

## DISCLOSURE OF AI USE

We acknowledge the use of ChatGPT, an AI language model developed by OpenAI, in refining the language and clarity of this case report manuscript. The AI was used to enhance readability, improve grammar, and ensure coherence while maintaining the integrity and originality of the content. All intellectual contributions, interpretations, and conclusions remain those of the authors.

## COMPETING INTEREST

The authors have no conflicts of interest to declare.
